# Electrophysiological and pathological changes in the vastus medialis and vastus lateralis muscles after early patellar reduction and nerve growth factor injection in rabbits with patellar dislocation

**DOI:** 10.1186/s13018-022-03170-w

**Published:** 2022-05-15

**Authors:** Yu Wu, Weifeng Li, Shiyu Tang, Changli Liu, Gang Ji, Fei Wang

**Affiliations:** Department of Orthopaedic Surgery, Third Hospital of Hebei Medical University, No. 139 Ziqiang Road, Shijiazhuang, 050051 Hebei China

**Keywords:** Patellar dislocation, Electrophysiological, Proprioceptor, Nerve growth factor (NGF)

## Abstract

**Background:**

Patellar dislocation can cause a series of changes in the trochlear groove and patella. However, the influence of patellar dislocation on the medialis (VM) and vastus lateralis (VL) muscles and whether nerve growth factor (NGF) is beneficial to proprioceptive rehabilitation for patellar dislocation are unknown. The purpose of this study was to investigate the effects on VM and VL after the injection of NGF and early reduction in rabbits for patellar dislocation with electrophysiological and pathological analysis.

**Methods:**

Sixty 2-month-old rabbits were randomly divided into four groups (15 rabbits in each group). Rabbits in Group 1, Group 2, and Group 3 underwent patellar dislocation surgery, and rabbits in Group 4 underwent sham surgery. One month later, patellar reduction was performed in Groups 1 and 2. NGF was injected into the rabbits of Group 1. The electrophysiological and pathological changes in VM and VL were analyzed at 1 month and 3 months after patellar reduction.

**Results:**

The electrophysiological and pathological indices in Groups 1 and 2 were significantly different from those in Group 3 at 1 and 3 months after patellar reduction. There were significant differences between NGF injection Group 1 and Group 2 without NGF injection. There was no significant difference between Group 1 and Group 4 at 3 months after patellar reduction.

**Conclusions:**

Patellar dislocation can cause abnormal electrophysiological and pathological effects on VM and VL. Patellar reduction should be performed as early as possible, and NGF injection may be beneficial to the rehabilitation of proprioception.

## Background

Patellar dislocation is a multifactorial clinical problem with an incidence of approximately 7 in 100,000 of the general population, and the rate is higher for younger and more active populations [1]. Patellar dislocations account for 3% of all knee injuries. The majority of injuries and pathology occur in young individuals. In particular, most patients with patellar instability are 10–16 years old [2]. Studies have shown that patellar dislocation can lead to a series of changes in the patellofemoral joint. The effect of the position of the patella on the development of the femoral trochlea and patella has been reported. Li et al. [3] and Wang et al. [4] found femoral trochlear dysplasia or flattening after patellar instability in growing rabbits. Niu et al. [5] found that the sectional shape and articular surface of the patella became more flattened after patellar dislocation, which indicated that patellar dysplasia could be caused by patellar instability. However, the patellofemoral joint is a whole structure and should be studied in an integrated manner. The effect of patellar dislocation on the electrophysiology and pathology of VL and VM is rarely reported.

Patellar dislocation can be caused by a number of factors. The stability of the patella depends on the force line of the lower limb, the shape of the femoral trochlea and patella, the integrity of the surrounding support ligament, and the interaction of the muscles around the patella. Under the condition of normal osseous structure of the knee joint, only correct adjustment of soft tissue balance can achieve satisfactory clinical effects. Therefore, a deep understanding of the internal and lateral soft tissues of the patella is the basis for selecting a reasonable surgical method to obtain satisfactory clinical efficacy. Experiments have shown that imitated complete loss of function of the VM will lead to minimum patellar stability when knee flexion reaches 30° [6]. Goh et al. [7]'s study also showed that changing the force distribution of the quadriceps femoris maximizes the displacement of the patella. The VM and VL play an important role in muscle balance in patella stability [8]. It is particularly important to study the VM and VL for a comprehensive understanding of patellar dislocation.

Niu et al. [9] and Wang et al. [4]'s studies indicated that early reduction could minimize the occurrence of femoral trochlear and patellar dysplasia in growing rabbits with patellar dislocation. But, the electrophysiological and pathological effects of patellar dislocation on VM and VL have not been confirmed. It is important to document the imbalance in muscle between VM and VL. Proprioception has been reported to be composed of incoming and outgoing pathways of somatosensory systems that control reflexes and muscle tone, which regulate the preciseness of the articular angles of the knee [10]. The VM and VL are directly connected to the patella and participate in proprioception. NGF is a neurotrophic factor released by mast cells, lymphocytes, and monocytes/macrophages in response to tissue inflammation and nociception [11]. Although NGF is a small secreted protein, it plays an irreplaceable role in the function and proliferation of nerve cells [12]. He et al. [13] revealed that NGF stimulates nerve regeneration by its biological activities on both neuronal and nonneuronal cells. In this study, we investigated the effects of NGF on proprioceptors in patellar dislocation. There are many methods for testing knee proprioception in the human body, but few for experimental animals. Somatosensory evoked potentials (SEPs), electromyography (EMG), and pathology are currently recognized as methods that can be used to check the state of knee proprioception in experimental animals [14]. The aim of this study was to investigate the role of patellar reduction and NGF in the proprioceptive system following patellar dislocation rehabilitation, and assess changes in the electrophysiology as well as the morphology and quantities of proprioceptors in growing rabbits after patellar dislocation.

## Methods

### Study design

The experimental protocol was approved by the Academic Ethics Committee of the Third Hospital of Hebei Medical University.

**Fig. 1:**
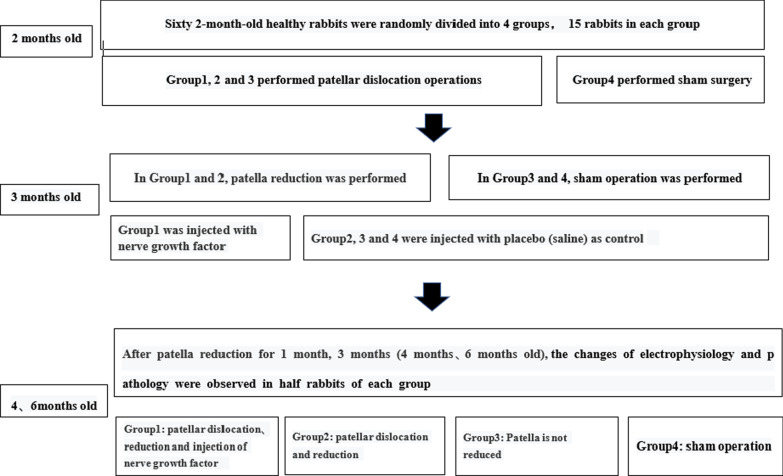
Schematic of the experimental protocol

Sixty New Zealand white rabbits, 2-month-old, weighing 460–560 g, female, were provided by the Laboratory Animal Center of Hebei Medical University. Preoperative examination of knee motion and gait of all young rabbits showed no abnormalities. The left knees of all the young rabbits were selected for surgery. The rabbits were randomly divided into four groups (15 in each group) according to the surgical method. Group 1: patellar dislocation was performed at 2 months old, and patellar reduction was performed with intramuscular injection of NGF 1 month later; Group 2: patellar dislocation was performed at 2 months, and patellar reduction was performed 1 month later; Group 3: patellar dislocation was performed at 2 months, and the patella was not reduced; and Group 4: sham operation group. The electrophysiological and pathological changes of the rabbits in each group were observed at 1 and 3 months after patellar reduction (Fig. 1). The previous literature has reported that rabbits tend to mature at the age of 6 months [15]; therefore, the observation time was controlled at 3 months post-surgery of patellar reduction when they were mature after the last follow-up.

### Surgical procedures

All surgeries were performed under intravenous anesthesia, ketamine (20 mg/kg), and xylazine (5 mg/kg). The rabbits were fixed on the platform for surgery with spine position. Before surgery, following standard protocol, the left lower limb was shaved and sterilized.

**Fig. 2:**
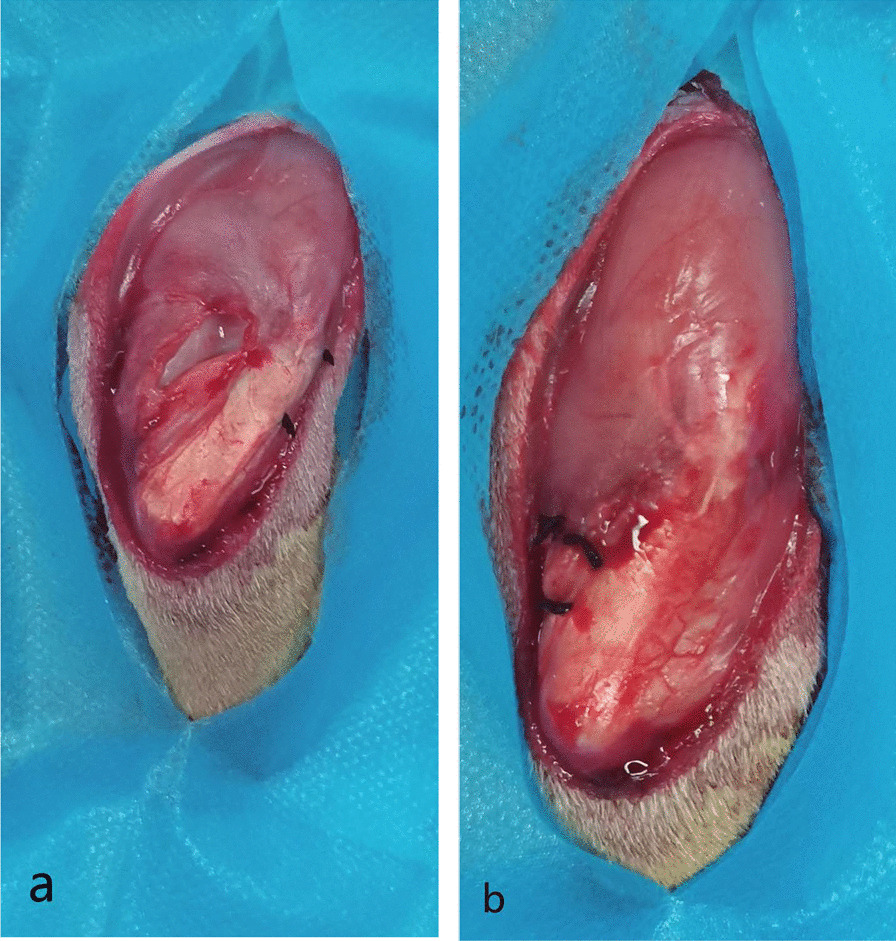
**a** At the insertion point of the femur, the medial retinacular band and joint capsule were cut, and the lateral joint capsule and lateral retinacular band were overlapped with two stitches. **b** The lateral joint capsule and lateral retinacular band were loosened, and the medial retinacular band and joint capsule were sutured to reduce the patella and kept in the trochlear tract

Most animal models of patellar dislocation were achieved by surgical releasing of medial patellofemoral retinacular band and further suturing with overlapping the lateral patellofemoral retinacular band [16]. This experiment also follows this principle. Furthermore, considering that the VM is directly connected to the patella, the medial patellofemoral retinacular band was cut at the insertion point of the femur, in order to avoid damaging the VM. A 3.5-cm incision was performed on the midline of knee skin; the soft tissue was dissected to expose the patellofemoral medial retinaculum and the joint capsule. For Group 4, the wound was closed at this point. At the insertion point of the femur, the medial retinacular band and joint capsule were incised about 2 cm, then the patella was pushed laterally to expose the femoral trochlea, and the later joint capsule and lateral retinaculum were overlapped and sutured together (Fig. 2a). At last, the incision was sutured.

Rabbits in Group 1 and Group 2 underwent patellar reduction at 1 month after patellar dislocation surgery via the original midline incision. The skin and soft tissues were dissected to expose the patella and its medial, lateral retinaculum. The lateral joint capsule and lateral retinacular band were loosened, and the medial retinacular band and joint capsule were sutured to reduce the patella and maintained in the femoral trochlear groove (Fig. 2b). As a control, in Group 3 and Group 4, the skin and subcutaneous tissue were incised and sutured. To avoid blood vessel and cartilage damage, the procedures were performed carefully. After patellar reduction, an intramuscular injection of nerve growth factor (20 μg/day, 202103002, Wuhan Hiteck Biopharmaceutical Co. Ltd., China) was initiated in Group 1 for 1 month, and intramuscular injection of normal saline was used as a control in the other three groups. Half of the rabbits were randomly selected from each of the four groups at 1 month, and the others were randomly selected for 3 months. SEPs and EMG were performed for electrophysiological examination. Accordingly, the rabbits were euthanized via a sodium pentobarbital overdose, and then the patellar insertion of VL and VM of the rabbits was removed for HE staining and immunohistochemical staining. The rabbits were raised in the same conditions. Each animal was housed in an individual stainless steel (310 × 550 × 320 mm) cage, which was sufficiently large, and the rabbits were allowed to move freely post-surgery. Based on their gait, the rabbits walked unsteadily on their left limbs, and their mobility was decreased slightly. Oral ciprofloxacin (10 mg/kg) was administered as antibiotic prophylaxis for the first 3 days post-surgery. The healing of the wound was achieved Grade A after patellar dislocation in rabbits.

### Electrophysiological analysis

Electrophysiological changes were assessed by EMG and SEPs at 1 and 3 months after patellar reduction. First, for the EMG examination procedure, the head and limbs were fixed after intravenous anesthesia with ketamine (20 mg/kg) and xylazine (5 mg/kg). Routine disinfection was performed, and an original midline incision was used to expose the VM and VL. The recording electrode was placed in the belly of the VM, and a reference electrode was inserted in front of the knee joint. The bipolar electrode was placed at the exit of the groin of the femoral nerve. The stimulation parameters were constant, the wave width was 0.2 ms, and the frequency was 1 HZ. EMG of VL was performed in the same way. Then, SEPs were tested, and the recording electrode was placed between the posterior edge of the two orbitals and 0.3–0.4 cm from the median sagittal line, which was the sensory area of the hind limb. The reference electrode was placed at the proximal insertions, and the electrode proximal to the lower limb was earthed. The 20 V stimulation intensity was constant with a unilateral wave stimulation of 0.1 ms. This data measurement in the electrophysiological analysis was carried out by one experienced physician. The information was recorded in the microcomputer operating system.

### Histopathological analysis

The VL and VM tissues were cut into 0.5–0.5 cm tissue blocks, fixed with paraformaldehyde, dehydrated, and embedded. Each tissue block was sliced along the long axis of the specimen on a microtome with a thickness of 10 μm and an interval of 150 μm. Four discontinuous sections were taken, two for HE staining and two for immunofluorescence staining. HE staining was followed by dewaxing, hematoxylin staining, dehydration, sealing, and other steps to make HE slices. Afterward, immunohistochemical staining, dewaxing, antigen repair, peroxidase removal, and serum sealing were performed. The primary antibody (S100) and secondary antibody (biotin-labeled goat anti-rabbit IgG) were removed, followed by DAB staining, retention, and dehydration sealing. The sections with HE staining and immunohistochemical staining were analyzed and photographed by the same observer under an optical microscope. The numbers of proprioceptors were counted in per cross-sectional area with a thickness of 10 μm. The pathological observers did not know which group each sample was from and were blinded at the time of the evaluation.

### Statistical analysis

The experimental data were expressed as the mean ± SD, and SPSS 23.0 software was used for statistical analysis. The Shapiro–Wilk (S-W) test was used for normal tests, one-way ANOVA was used for comparisons between groups, and an independent sample t test was used for comparisons between different periods. *P* < 0.05 was considered as statistically significant.

## Results

**Fig. 3:**
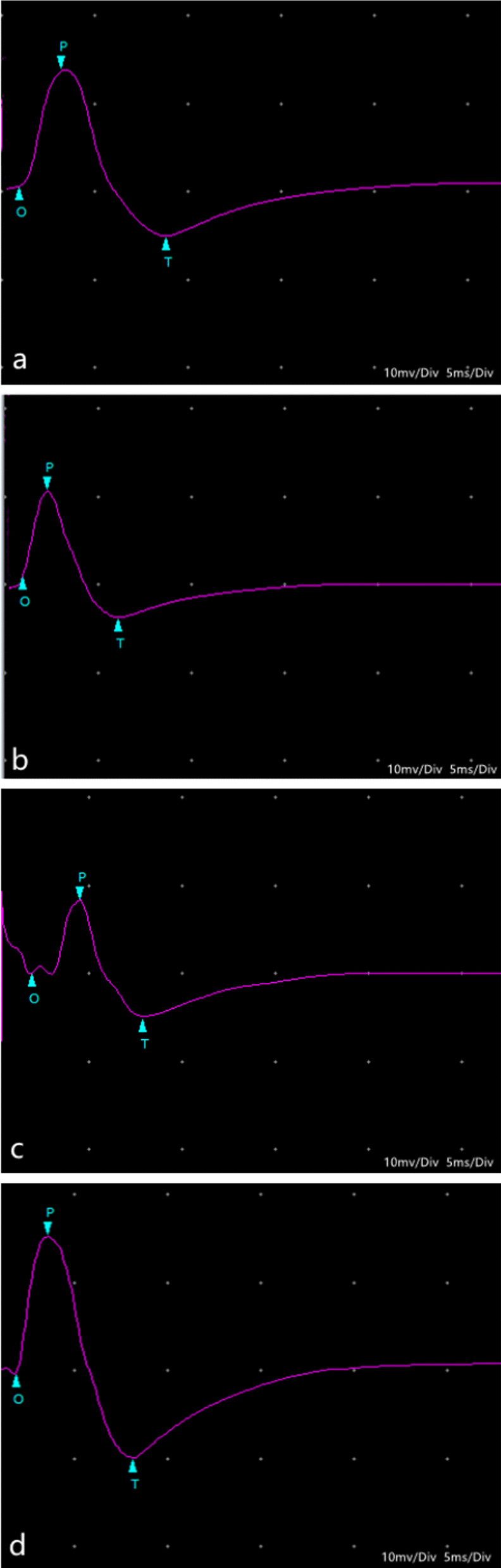
VM EMG images at 3 months after patella reduction. **a** Group 1. **b** Group 2. **c** Group 3. **d** Group 4

**Fig. 4:**
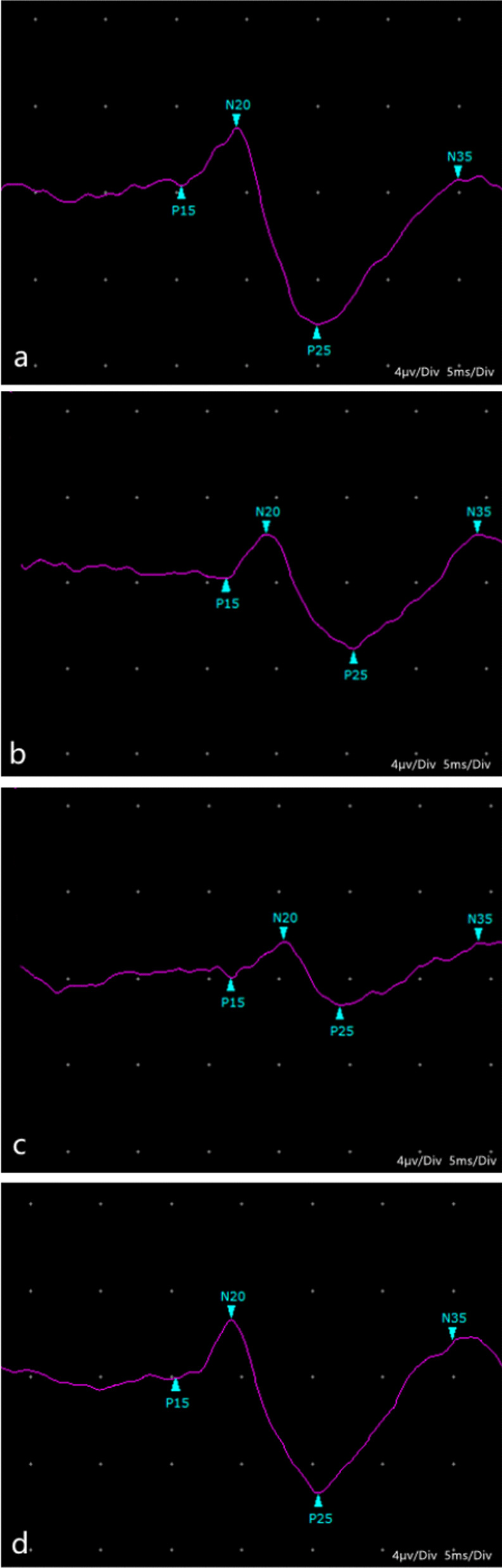
VM SEPs images at 3 months after patella reduction. **a** Group 1. **b** Group 2. **c** Group 3. **d** Group 4

**Fig. 5:**
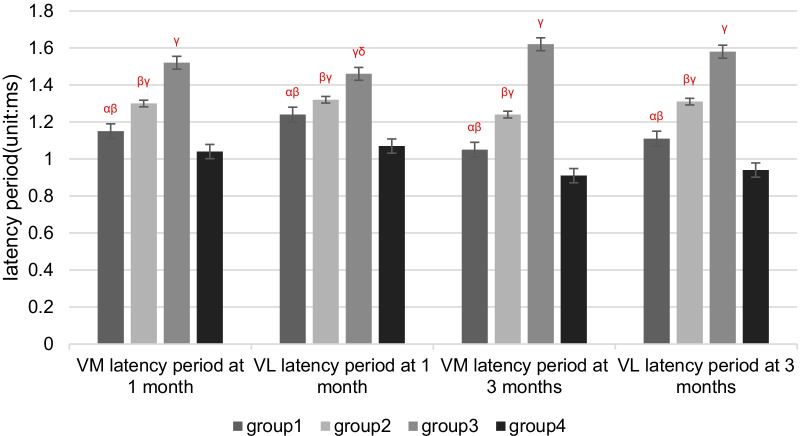
Four groups regarding the latency period of EMG at different detection times. *α* *P* < 0.05 versus Group 2 in the same month; *β* *P* < 0.05 versus Group 3 in the same month; *γ* *P* < 0.05 versus Group 4 in the same month; *δ* *P* < 0.05 versus 3 months in the same group

**Fig. 6:**
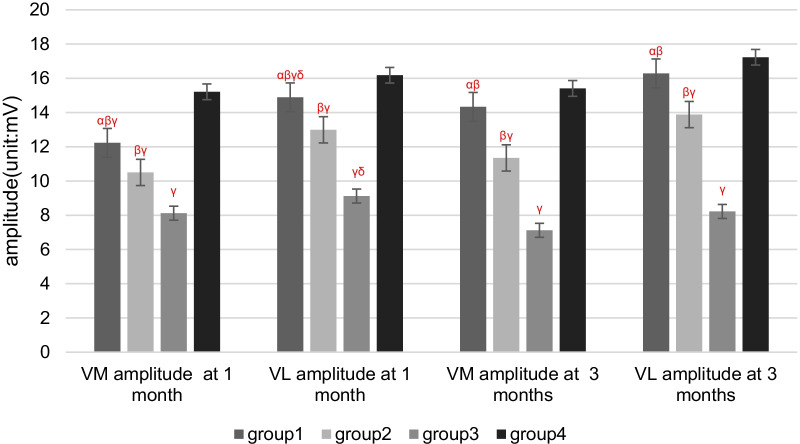
Four groups regarding the amplitude of EMG at different detection times. *α* *P* < 0.05 versus Group 2 in the same month; *β* *P* < 0.05 versus Group 3 in the same month; *γ* *P* < 0.05 versus Group 4 in the same month; *δ* *P* < 0.05 versus 3 months in the same group

**Fig. 7:**
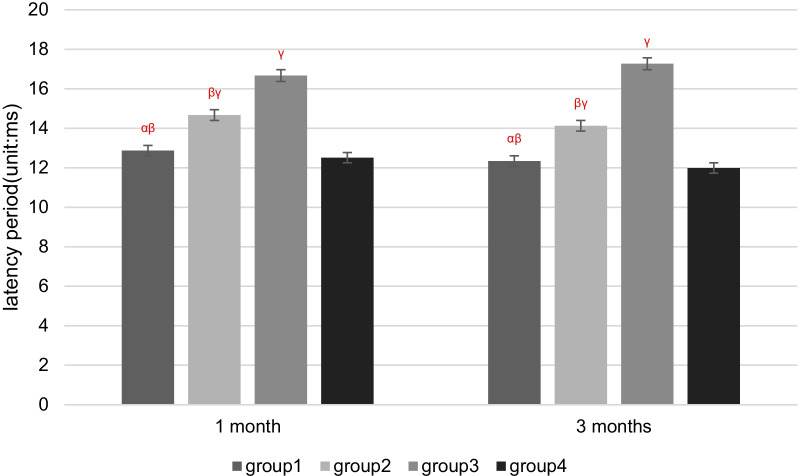
Four groups regarding the latency period of SEPs at different detection times. *α* *P* < 0.05 versus Group 2 in the same month; *β* *P* < 0.05 versus Group 3 in the same month; *γ* *P* < 0.05 versus Group 4 in the same month; *δ* *P* < 0.05 versus 3 months in the same group

**Fig. 8:**
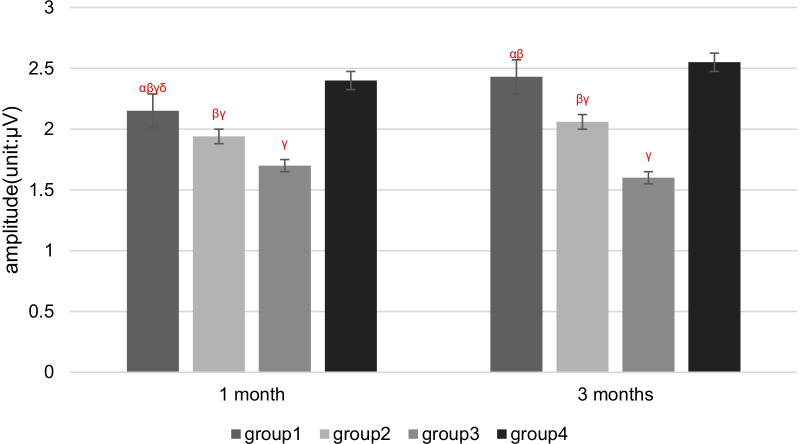
Four groups regarding the amplitude of SEPs at different detection times. *α* *P* < 0.05 versus Group 2 in the same month; *β* *P* < 0.05 versus Group 3 in the same month; *γ* *P* < 0.05 versus Group 4 in the same month; *δ* *P* < 0.05 versus 3 months in the same group

Two rabbits died of diarrhea in 1 month after patellar reduction surgery. Therefore, 58 rabbits were used for electrophysiological and pathological tests. There were no cases where patella reduced by itself after dislocation surgery. There were no cases of patella dislocation after reduction. Compared with Groups 1 and 2, the latency periods were extended, and the amplitudes of SEPs and EMG were decreased in Group 3 at 1 and 3 months after patellar reduction, indicating that patellar reduction was beneficial to electrophysiological rehabilitation. The SEPs and EMG in Group 1 were not significantly different from those in Group 4 at 3 months after patellar reduction (all *P* values > 0.05. EMG: amplitude of VM, *P* = 0.144; latency period of VM, *P* = 0.083; amplitude of VL, *P* = 0.099; latency period of VL, *P* = 0.059. SEPs: amplitude, *P* = 0.082; latency period, *P* = 0.68). The amplitudes at 3 months after patellar reduction were significantly higher than those at 1 month in Group 1. Moreover, the amplitudes were decreased, and the latency periods were extended for SEPs and EMG in Group 2 compared with Group 1 at the same time point (all *P* values < 0.05. EMG (1 month): amplitude of VM, *P* = 0.04; latency period of VM, *P* = 0.04; amplitude of VL, *P* = 0.01; latency period of VL, *P* = 0.01. EMG (3 months): amplitude of VM, *P* = 0.00; latency period of VM, *P* = 0.02; amplitude of VL, *P* = 0.00; latency period of VL, *P* = 0.04. SEPs (1 month): amplitude, *P* = 0.03; latency period, *P* = 0.02. SEPs (3 months): amplitude, *P* = 0.00; latency period, *P* = 0.02) (Figures 3 and 4). These results indicate that NGF can effectively shorten the latency period and increase the amplitude (Tables 1, 2; Figs. 5, 6, 7, 8).

**Table 1:** Comparison among the four groups regarding the latency period and amplitude of EMG at different detection times

Index	Group 1		Group 2		Group 3		Group 4	
	Latency period (ms)	Amplitude (mV)	Latency period (ms)	Amplitude (mV)	Latency period (ms)	Amplitude (mV)	Latency period (ms)	Amplitude (mV)
*1 month*								
VM	1.15 ± 0.07^αβ^	12.23 ± 2.12^αβγ^	1.30 ± 0.17^βγ^	10.50 ± 1.13^βγ^	1.52 ± 0.13^γ^	8.12 ± 0.95^γ^	1.04 ± 0.12	15.21 ± 1.47
VL	1.24 ± 0.15^αβ^	14.89 ± 1.19^αβγδ^	1.32 ± 0.14^βγ^	12.99 ± 1.29^βγ^	1.46 ± 0.09^γδ^	9.12 ± 0.30^γδ^	1.07 ± 0.12	16.18 ± 1.45
*3 months*								
VM	1.05 ± 0.16^αβ^	14.33 ± 1.94^αβ^	1.24 ± 0.18^βγ^	11.35 ± 0.88^βγ^	1.62 ± 0.14^γ^	7.12 ± 1.33^γ^	0.91 ± 0.12	15.41 ± 1.44
VL	1.11 ± 0.91^αβ^	16.29 ± 1.17^αβ^	1.31 ± 0.21^βγ^	13.88 ± 1.24^βγ^	1.58 ± 0.94^γ^	8.22 ± 0.36^γ^	0.94 ± 0.16	17.23 ± 1.31

*α* *P* < 0.05 versus Group 2 in the same month; *β* *P* < 0.05 versus Group 3 in the same month; *γ* *P* < 0.05 versus Group 4 in the same month; *δ* *P* < 0.05 versus 3 months in the same group

**Table 2:** Comparison among the four groups regarding the latency period and amplitude of SEPs at different detection times

Index	Group 1		Group 2		Group 3		Group 4	
	Latency period (ms)	Amplitude (μV)	Latency period (ms)	Amplitude (μV)	Latency period (ms)	Amplitude (μV)	Latency period (ms)	Amplitude (μV)
1 month	12.87 ± 0.81^αβ^	2.15 ± 0.10^αβγδ^	14.67 ± 1.40^βγ^	1.94 ± 0.20^βγ^	16.67 ± 1.36^γ^	1.70 ± 0.15^γ^	12.51 ± 1.80	2.40 ± 0.21
3 months	12.34 ± 0.79^αβ^	2.43 ± 0.13^αβ^	14.13 ± 1.40^βγ^	2.06 ± 0.18^βγ^	17.27 ± 1.36^γ^	1.60 ± 0.12^γ^	11.99 ± 1.85	2.55 ± 0.22

*α* *P* < 0.05 versus Group 2 in the same month; *β* *P* < 0.05 versus Group 3 in the same month; *γ* *P* < 0.05 versus Group 4 in the same month; *δ* *P* < 0.05 versus 3 months in the same group

**Fig. 9:**
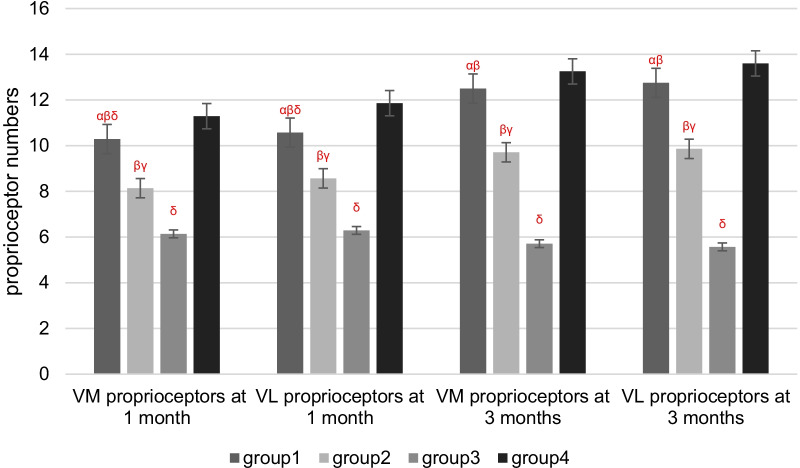
Four groups regarding the number of proprioceptors at different detection times. *α* *P* < 0.05 versus Group 2 in the same month; *β* *P* < 0.05 versus Group 3 in the same month; *γ* *P* < 0.05 versus Group 4 in the same month; *δ* *P* < 0.05 versus 3 months in the same group

**Fig. 10:**
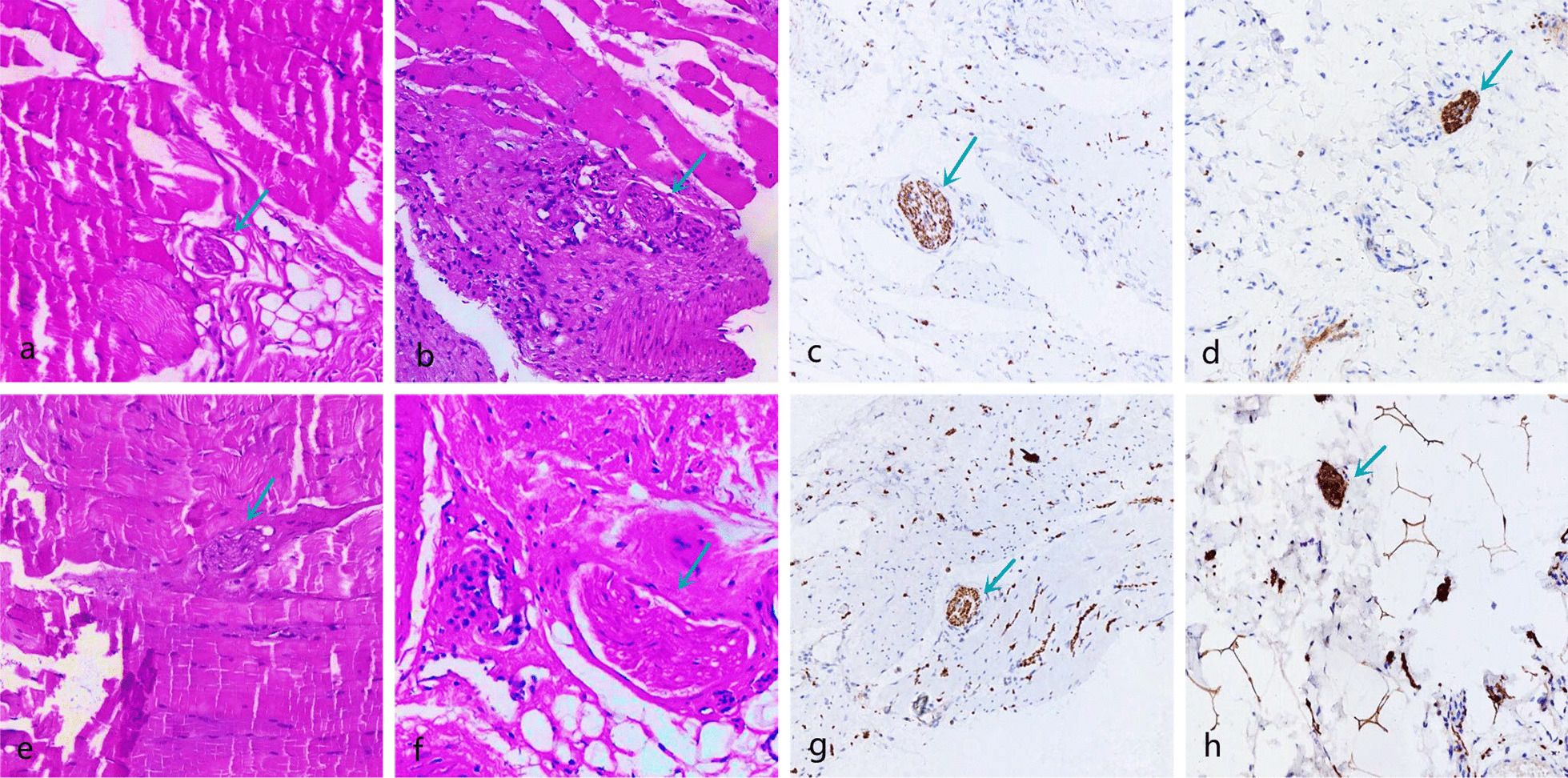
Pathology of Group 1; the green arrow refers to the proprioceptors. The morphology was basically normal, and the membrane was dense without obvious atrophied or deformed. **a**, **b** VM and VL (HE × 100) at 1 month after patella reduction; **c**, **d** VM and VL (immunofluorescence × 100) at 1 month after patella reduction. **e**, **f** VM and VL (HE × 100) at 3 months after patella reduction; **g**, **h** VM and VL (immunofluorescence × 100) at 3 months after patella reduction

**Fig. 11:**
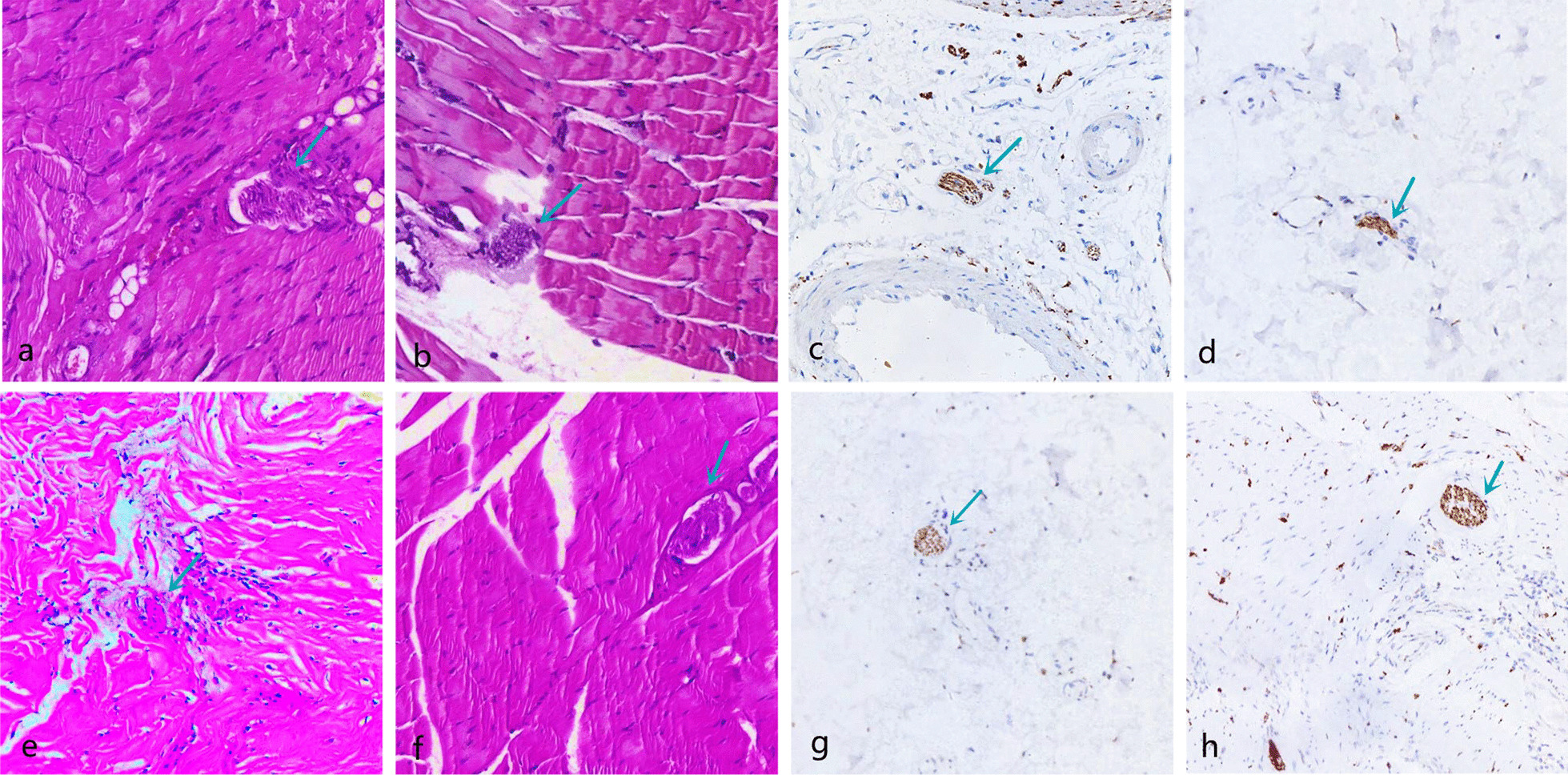
Pathology of Group 2; the green arrow refers to the proprioceptors. The proprioceptors were widened and arranged loosely, and the outer thin-walled connective tissue sac changed irregularly. **a**, **b** VM and VL (HE × 100) at 1 month after patella reduction; **c**, **d** VM and VL (immunofluorescence × 100) at 1 month after patella reduction. **e**, **f** VM and VL (HE × 100) at 3 months after patella reduction; **g**, **h** VM and VL (immunofluorescence × 100) at 3 months after patella reduction

**Fig. 12:**
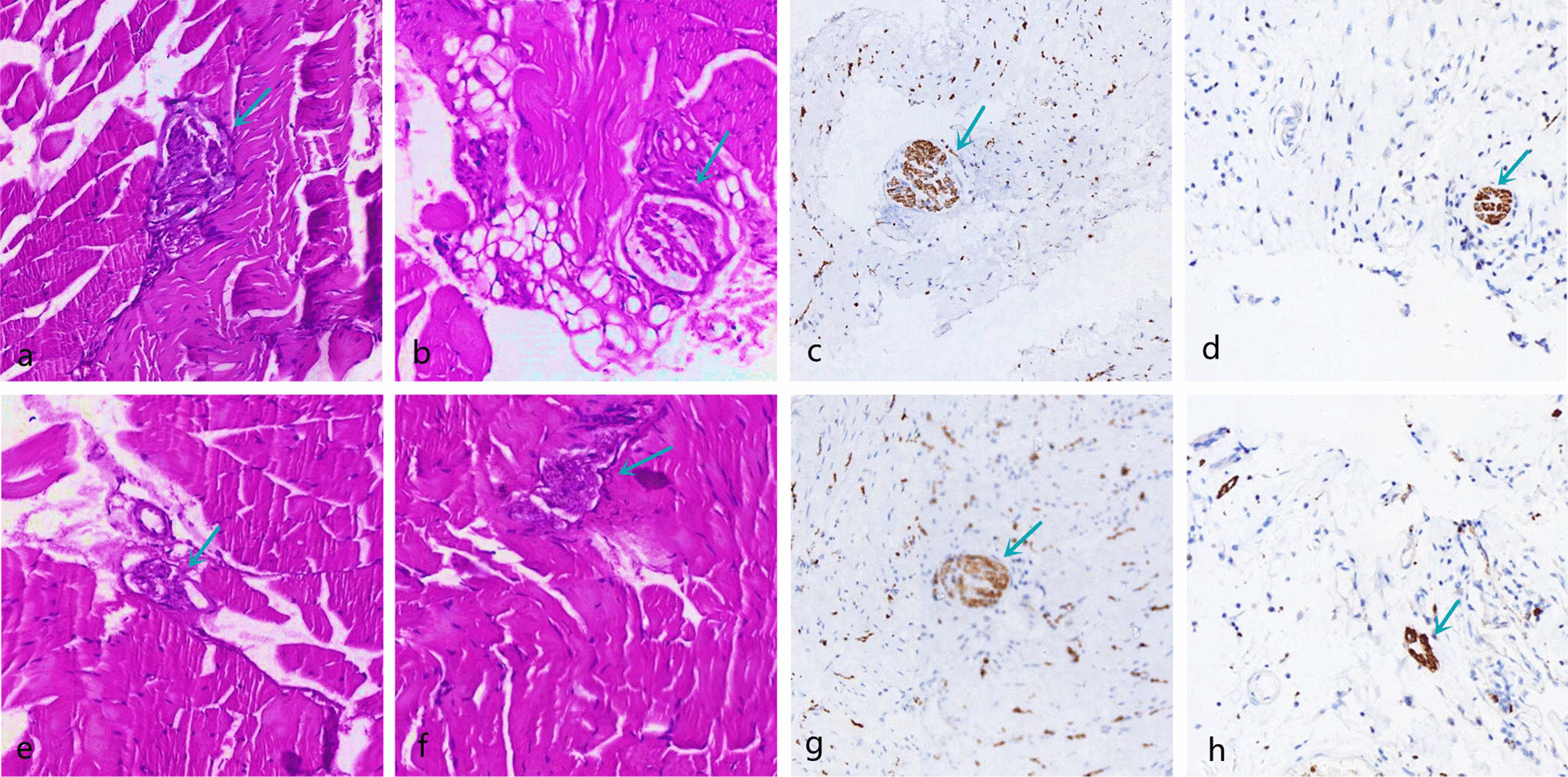
Pathology of Group 3; the green arrow refers to the proprioceptors. The proprioceptors were arranged in disorder and loose, the long axis direction from parallel to irregular, atrophied, deformed, and cleavage. **a**, **b** VM and VL (HE × 100) at 1 month after patella reduction; **c**, **d** VM and VL (immunofluorescence × 100) at 1 month after patella reduction. **e**, **f** VM and VL (HE × 100) at 3 months after patella reduction; **g**, **h** VM and VL (immunofluorescence × 100) at 3 months after patella reduction

**Fig. 13:**
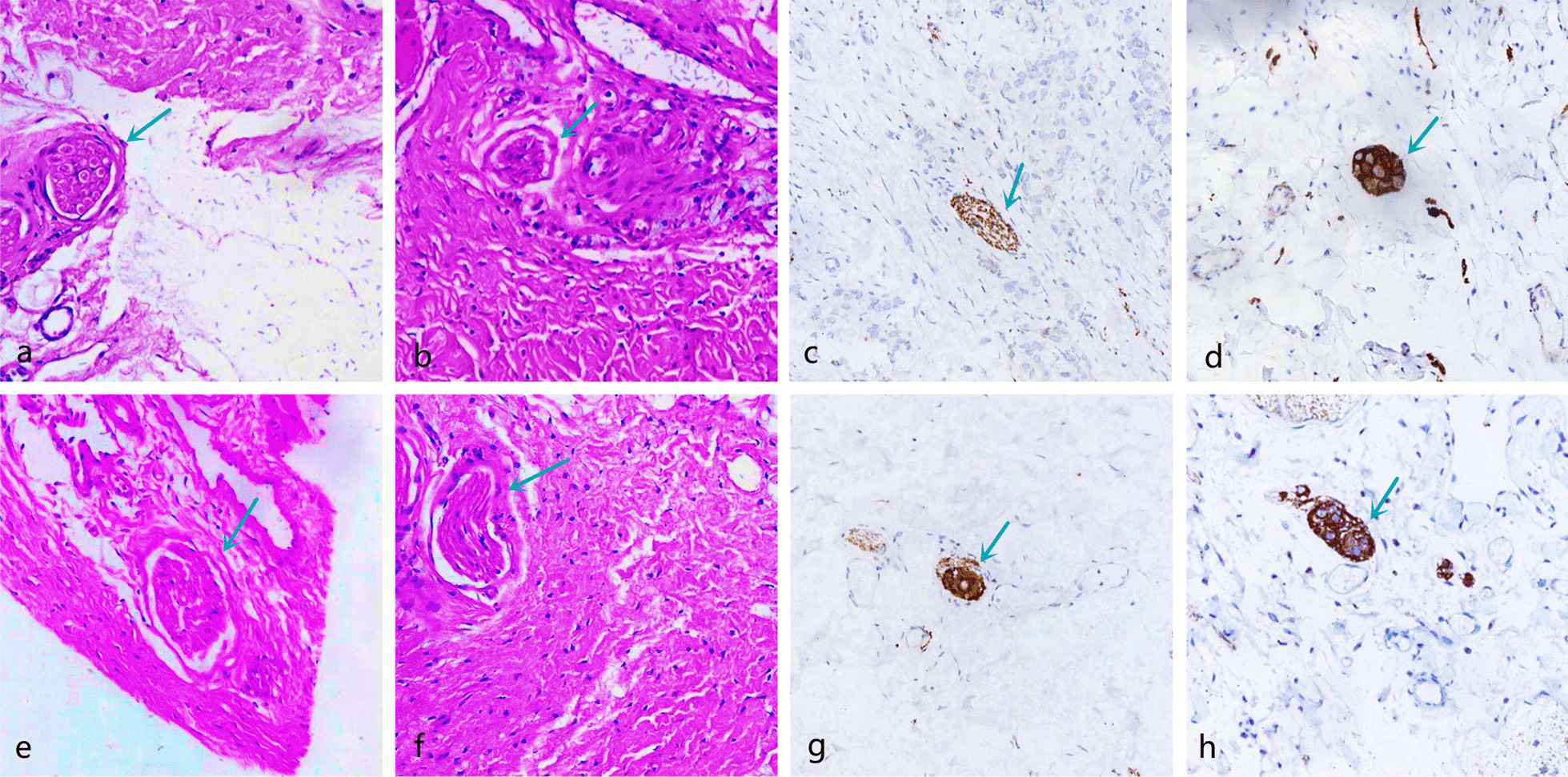
Pathology of Group 4; the green arrow refers to the proprioceptors. The proprioceptors were round or oval with regular shapes and smooth edges. **a**, **b** VM and VL (HE × 100) at 1 month after patella reduction; **c**, **d** VM and VL (immunofluorescence × 100) at 1 month after patella reduction. **e**, **f** VM and VL (HE × 100) at 3 months after patella reduction; **g**, **h** VM and VL (immunofluorescence × 100) at 3 months after patella reduction

The morphology of proprioceptors was recorded after HE and immunofluorescence staining (Table 3; Figs. 9, 10, 11, 12, 13). The number of proprioceptors in Group 3 was significantly decreased compared with that in Groups 1 and 2 at 1 and 3 months after patellar reduction. The morphology of proprioceptors was significantly atrophied and deformed, indicating that patellar dislocation could lead to proprioceptor dysplasia. In comparison with Group 2, the number of proprioceptors in Group 1 was increased at both 1 and 3 months after patellar dislocation, and the changes were significant (all *P* values < 0.05. 1 month: the number of VM proprioceptors, *P* = 0.01; the number of VL proprioceptors, *P* = 0.01. 3 months: the number of VM proprioceptors, *P* = 0.00; the number of VL proprioceptors, *P* = 0.00). However, the difference in proprioceptor numbers between Group 1 and Group 4 was not significant (all *P* values > 0.05. 1 month: the number of VM proprioceptors, *P* = 0.20; the number of VL proprioceptors, *P* = 0.09. 3 months: the number of VM proprioceptors, *P* = 0.31; the number of VL proprioceptors, *P* = 0.33), suggesting that NGF could promote the regeneration of proprioceptors.

**Table 3:** Comparison among the four groups regarding the number of proprioceptors at different detection times

Index	Group 1	Group 2	Group 3	Group 4
*1 month*				
VM	10.29 ± 1.25^αβδ^	8.14 ± 1.22^βγ^	6.14 ± 1.22^δ^	11.29 ± 1.89
VL	10.57 ± 1.27^αβδ^	8.57 ± 1.27^βγ^	6.29 ± 1.11^δ^	11.86 ± 1.77
*3 months*				
VM	12.50 ± 1.20^αβ^	9.71 ± 1.50^βγ^	5.71 ± 1.38^δ^	13.25 ± 1.67
VL	12.75 ± 1.49^αβ^	9.86 ± 1.35^βγ^	5.57 ± 1.51^δ^	13.60 ± 1.60

*α* *P* < 0.05 versus Group 2 in the same month; *β* *P* < 0.05 versus Group 3 in the same month; *γ* *P* < 0.05 versus Group 4 in the same month; *δ* *P* < 0.05 versus 3 months in the same group

## Discussion

The most important finding of this study was that patellar dislocation could cause changes in the electrophysiology and proprioceptors of VM and VL. Patellar reduction was beneficial to the rehabilitation of VM and VL. Moreover, NGF injection had a significant effect on the electrophysiology and pathology of muscle. Most of the studies on the restoration of knee proprioception focus on the basis of physical rehabilitation exercise, but the effect is not satisfactory. Although the biomechanical stability of the patellofemoral joint is irreplaceable, more attention is paid to proprioception and maintaining the dynamic stability of the normal patellar trajectory. It has been reported that proprioception is involved in the regulation of motion by the human nervous system [17], which is correlated with natural sensation of the knee joint [18].

Proprioception of the knee joint plays an important clinical role in controlling joint stability, correcting body posture, and maintaining body balance; proprioception of the knee joint mainly comes from the muscles around the joint [19]. Proprioceptive stress stimulation can make the body respond to joint stress faster and reduce the rate of joint wear and dislocation [20]. This study showed that after patellar dislocation, the mechanical proprioceptors of VM and VL were atrophied, deformed, and decreased (Group 3 data in Table 3). Proprioceptors were increased after patellar reduction (Group 1 and Group 2 data in Table 3). Mechanical stress is one of the most important factors affecting growth [21]. It is considered to be related to the loss of normal stress stimulation and VM and VL activity after patellar dislocation in growing rabbits. Specific immunohistochemical staining and microscopic observation of VM and VL in rabbits were conducted in this study. The distribution, types, quantity, and morphology of proprioceptors were then analyzed. The results suggested that patellar dislocation should be corrected as early as possible to minimize the occurrence of secondary changes.

The purpose of patellar reduction surgery is to stabilize the patella in the trochlear groove and strengthen the tension of the VM. Smillie [22] referred to the VM as the “key to the knee” to illustrate its importance in patellar stabilization. SEPs and EMG have been widely accepted as methods to detect the sensory state of knee joints in animals [14]. Statistically significant differences in electrophysiology and pathology in the reduction group and patellar dislocation group were noted. The reason may be that the flexion and extension of the quadriceps muscle stimulate nerve proprioceptors. In contrast, the indices of Group 3 were worse at the age of 6 months than at 4 months, highlighting the importance of maintaining the normal patellar trajectory. Any changes in muscle balance between the VM and VL can affect the patellofemoral joint, whether congenital or posttraumatic, depending on the severity of the disorder.

Proprioception is defined as the ability to perceive sensory stimuli such as touch, pain, pressure, and movement. Joint position sense and joint motion sense are common proprioception senses that enable individuals to accurately perceive joint position and motion. Proprioception is reported to play an important role in the regulation of the nervous system and motor performance [23]. The effects of joint proprioceptive information on motion control can be divided into two categories [24]: first, the appropriate response of the body to external environmental changes. Through the central adjustment in joint motions to adapt to the change in the external environment, for example, walking should be performed according to the road surface's situation to change the pace and direction. Although most of the sensory information descendants are produced by the visual system and central nervous system, body sensory information is accurate and fastest and plays an irreplaceable role. Second, they participate in the integration of higher orders in the sensorimotor center. In the process of movement, the motor center regulates skeletal and muscle activities, and proprioceptive information contains the necessary joint position and strength information [25]. Proprioceptors, the vestibule, and vision work together to control muscles. The control process of motion requires continuous sensory integration and information input by the proprioceptors of muscles. The proprioceptor information procedure may be interrupted when injury occurs; as a result, the sensorimotor center cannot quickly and accurately perceive the position, movement, muscle force, and other information of the joint.

Surgical treatment of patellar dislocation has achieved good clinical results. Patients may benefit from prompt surgical intervention to repair soft tissue injuries and restore patellar tracking, preventing further redislocations [26]. In patients with patellar dislocation, the mechanical stability of the knee joint must be ensured, but attention should also be paid to joint proprioception. Drug intervention is a hot research topic in proprioception rehabilitation. NGF plays an important role in proprioceptive repair [27]. Among the members of the neurotrophic factor family, NGF was the first to be discovered and is collectively known as a neurotrophic factor [28]. As early as the 1950s, R. Levi-Montalcini carried out pioneering studies on laboratory animals and isolated cells, focusing on the biological role of NGF [29]. These studies suggest that NGF protects the survival of degraded or damaged peripheral nerve cells and has a protective effect on the regulation of neurotransmitter and neuropeptide synthesis [30]. After muscle injury or degeneration, NGF can reduce the expression of injury-induced transcription Factor 3 and promote the reneutralization of target organs [31], thereby protecting proprioceptive neurons. Morphological and quantitative changes in proprioceptors in the VM and VL were observed. The study found that after surgical reduction, in the NGF-injected group, the proprioceptors were different from those in the group without NGF injection with small size and partial structural loss, and the indices of SEPs and EMG were statistically significant. Treatment with NGF was beneficial to the rehabilitation of knee proprioception.

The dynamic position of the patella is dependent on the vectors of VM and VL [6]. The proprioceptors of VM and VL can collect information on joint movement and position, which can be transmitted to the spinal cord and brain through afferent nerves. The information is analyzed and integrated through the efferent nerve, and the corresponding response is made to adjust the motion and stability of the knee joint. Patellofemoral joint instability is mainly manifested by poor balance and coordination ability, significantly decreased spatial position judgment ability, poor nerve reactivity, prolonged nerve response to sudden torsional stress and electrical stimulation, and weakened muscle strength [32]. After a first episode of patellar dislocation, most patients presented with soft tissue lesions [33]. Electrophysiological examination is an important means to monitor muscle balance. Electrophysiological tests were performed on VM and VL to demonstrate muscle imbalance in patellar dislocation and the effectiveness of patellar reduction. VM can prevent lateral dislocation of the patella by resisting the pressure generated by knee valgus angulations and VL, which is central to the stabilization of the patella [22]. It is suggested that special attention should be given to functional exercise of the muscle after patellar dislocation, and at the same time, drugs promoting proprioception rehabilitation can be used to facilitate its treatment.

This study demonstrated that functional instability and mechanical instability were not distinct but related to each other, and their joint persistent action ultimately led to chronic instability of the joint. In this study, the proprioception of the patellar dislocation model in growing rabbits was significantly improved after surgical reduction. The results indicated that patellar reduction could improve functional stability as well as mechanical stability. Early reduction is conducive to the rehabilitation of VM and VL muscle coordination and balance and is thus conducive to the stability of the patella. Clinically, functional instability should be considered as well as mechanical stability in the treatment and rehabilitation of patients with patellar dislocation, which causes not only changes in skeletal tissue but also soft tissue imbalance. An integrated treatment should be further investigated, and attention should be given to electrophysiological changes in muscles around the knee joint.

There were several limitations to this study. First, rabbit models have been widely used to study patellar dislocation, but rabbit knees are bent and cannot be equated to upright walking humans. Second, patellar dislocation surgery was performed in rabbits at 2 months of age, which mimics early patellar dislocation in humans but does not completely coincide with the time point of patellar dislocation in humans. In addition, the number of rabbits in the experiment was relatively small, and although it was enough to be statistically significant, large numbers of animals would be better. Finally, the integrated treatment of patellar dislocation needs to be further studied.

## Conclusions

Based on the outcomes of the study, we conclude that patellar dislocation can lead to abnormal electrophysiological and pathological effects on VM and VL in growing rabbits. This study confirms that early reduction is necessary and NGF injection could improve joint function rehabilitation by promoting the quality and quantity of proprioceptors for patellar dislocation.

## Data Availability

The detailed data and materials of this study are available from the corresponding author via e-mail on reasonable request.

## Abbreviations

VM: Vastus medialis
VL: Vastus lateralis
EMG: Electromyography
SEPs: Somatosensory evoked potentials
NGF: Nerve growth factor
